# Superstretchable, yet stiff, fatigue-resistant ligament-like elastomers

**DOI:** 10.1038/s41467-022-30021-3

**Published:** 2022-04-27

**Authors:** Mengxue Li, Lili Chen, Yiran Li, Xiaobin Dai, Zhekai Jin, Yucheng Zhang, Wenwen Feng, Li-Tang Yan, Yi Cao, Chao Wang

**Affiliations:** 1 Key Lab of Organic Optoelectronics & Molecular Engineering, Department of Chemistry, Tsinghua University, Beijing, China; 2 Department of Physics, Nanjing University, Nanjing, China; 3 State Key Laboratory of Chemical Engineering, Department of Chemical Engineering, Tsinghua University, Beijing, 100084 China

**Keywords:** Mechanical properties, Mechanical properties, Polymers

## Abstract

Ligaments are flexible and stiff tissues around joints to support body movements, showing superior toughness and fatigue-resistance. Such a combination of mechanical properties is rarely seen in synthetic elastomers because stretchability, stiffness, toughness, and fatigue resistance are seemingly incompatible in materials design. Here we resolve this long-standing mismatch through a hierarchical crosslinking design. The obtained elastomer can endure 30,000% stretch and exhibit a Young’s modulus of 18 MPa and toughness of 228 MJ m^−3^, outperforming all the reported synthetic elastomers. Furthermore, the fatigue threshold is as high as 2,682 J m^−2^, the same order of magnitude as the ligaments (~1,000 J m^−2^). We reveal that the dynamic double-crosslinking network composed of Li^+^-O interactions and PMMA nanoaggregates allows for a hierarchical energy dissipation, enabling the elastomers as artificial ligaments in soft robotics.

## Abstract (web summary)

Stiffness, toughness, and fatigue resistance are seemingly incompatible in materials design. Here the authors demonstrate a hierarchical crosslinking strategy using lithium ion oxygen interactions and PMMA nanoaggregates to enable energy dissipation in the network, leading to stiff yet tough polymer materials.

## Introduction

The human ligaments are stiff, tough, elastic materials composed of collagen fibers, elastic fibers, and reticular fibers^1^. Collagen fibers provide strength and stiffness, elastic fibers provide stretchability, and reticular fibers provide volume. These three fibers synergistically contribute to the high stretchability, stiffness, toughness, and fatigue resistance of the ligaments. Synthetic ligament-like elastomers are highly desirable for soft robotics, flexible electronics, and biomedical applications, where the polymers are constantly subjected to dynamic loads^2–4^. But the combination of stretchability, stiffness, toughness, and fatigue resistance in the synthetic materials remains a challenge. According to the Lake-Thomas model in a covalently-bonded single network, as the number of monomer units per chain (*n*) increases, the threshold (*Γ*_*th*_) increase by Γ_*th*_ ∽ *n*^(1/2)^, the stiffness (*E*) decreases by *E* ∽ *n*^−1^, and according to the flexible chain theory, the maximum tensile ratio increases by *λ* ∽ *n*^(1/2)^^5,6^. Consequently, synthetic elastomers would not synergistically achieve all these properties. For example, the reported highest fatigue threshold of synthetic elastomers was 500 J m^−2^ (composite silicone rubber) with a low modulus of 0.1 MPa^7^.

Herein, we propose a new dynamic double-crosslinking design by integrating two types of dynamic bonds which differ in strength into a single polymer. On the one hand, we incorporate a kind of highly dynamic Li^+^-O ion-dipole interactions^8^ into the PEGA brush structure to dissipate most of the mechanical stress. On the other hand, we use rigid and loosely packed PMMA nanodomains^9,10^ to provide the stiffness and strength.

**Fig. 1:**
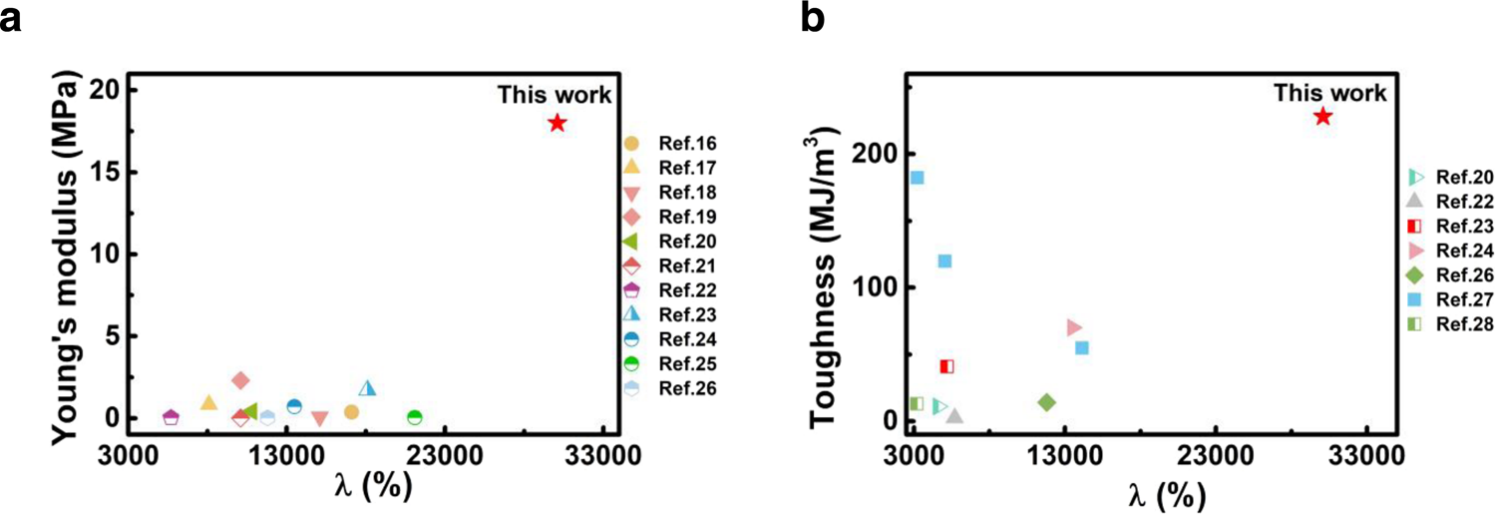
Summary of reported superstretchable, stiff and tough polymers. **a** Ashby plot of “Young’s modulus” and "*λ*" of this work and other stretchable materials (*λ* > 3,000%, *λ = L/L*_*0*_, *L*_*0*_ is the initial length between the clamps, *L* is total length between the clamps). **b** Ashby plot of “Toughness” and "*λ"* of this work and other stretchable materials (*λ* > 3,000%).

In contrast to previous energy-dissipating mechanisms with dynamic bonds^11–15^ in the polymer networks, the amorphous PMMA physical crosslinkers can disentangle at large stretchs, which requires additional energy to overcome the interfacial energy between PMMA nanodomains and soft PEGA parts, leading to significantly enhanced mechanical properties without the sacrifice of stretchability. As a result, the polymer achieves a superstretchability exceeding 30,000% with a Young’s modulus of 18 MPa, outperforming all the highly stretchable polymers (*λ* > 3000%) (Fig. 1a, Supplementary Table 1)^16–26^. And the high toughness (~228 MJ m^−3^) of our polymer is also incomparable in superstretchable materials (*λ* > 3000%) (Fig. 1b, Supplementary Table 2)^20,22–24,26–28^. Moreover, the polymer has a remarkable fatigue threshold as high as 2,682 J m^−2^ in synthetic elastomers^7,29,30^, on the same magnitude as the ligament^31^ (~1000 J m^−2^) (Supplementary Table 3).

## Results

### Polymer design

**Fig. 2:**
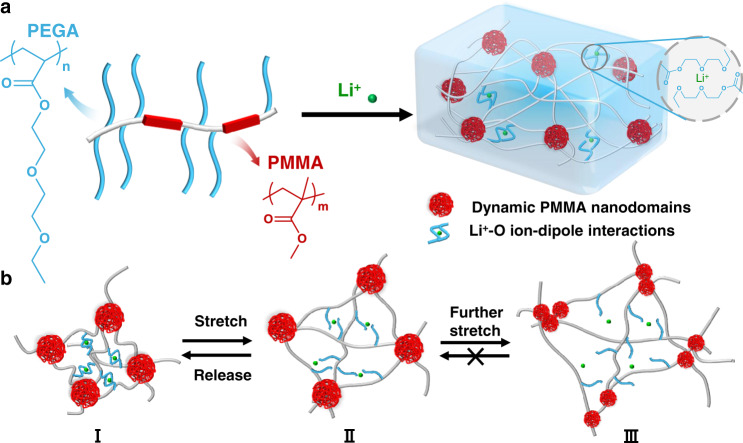
Structure design. **a** Chemical structure design and scheme of the double-crosslinking structure. **b** Proposed mechanism for bond breakage and reformation during tensile stretching: I. Initial II. At small strain, Li^+^-O weak bonds can easily break, but it can reform once the stress is removed. III. At large strain, PMMA nanodomains break into smaller parts to further dissipate energy.

We propose a methacrylate-acrylate-based copolymer, which consists of two components: a) soft brush-like PEGA blocks and b) rigid PMMA blocks (Fig. 2a). In this strategy, the weak crosslinking is provided by the ion-dipole interactions between lithium ions and the di(ethylene glycol) side chains of the PEGA blocks. With high binding affinity and dynamic nature, the multiple Li^+^-O ion-dipole interactions are expected to render the material simultaneous high elasticity and stretchability feature. The PMMA segments can aggregate into loose nanodomains and serve as stiff but dynamic crosslinkers benefitting from the volume repulsion brought by chiral carbons on the main chains. To be specific, whereas large-size PMMA domains are often intuitively considered as rigid and immovable polymers^10^, small-size PMMA packings can be disengaged especially under high mechanical stress (Fig. 2b)^9^. Besides the function of Li^+^-PEGA blocks for eliminating stress concentration and dissipating mechanical energy, strong and dynamic PMMA physical crosslinkers are indispensable for providing interfacial energy to resist the crack formation^32^. The double crosslinkers which differ in strength can achieve hierarchical dissipative ability for improving the toughness and fatigue threshold.

### High stretchability and stiffness

**Fig. 3:**
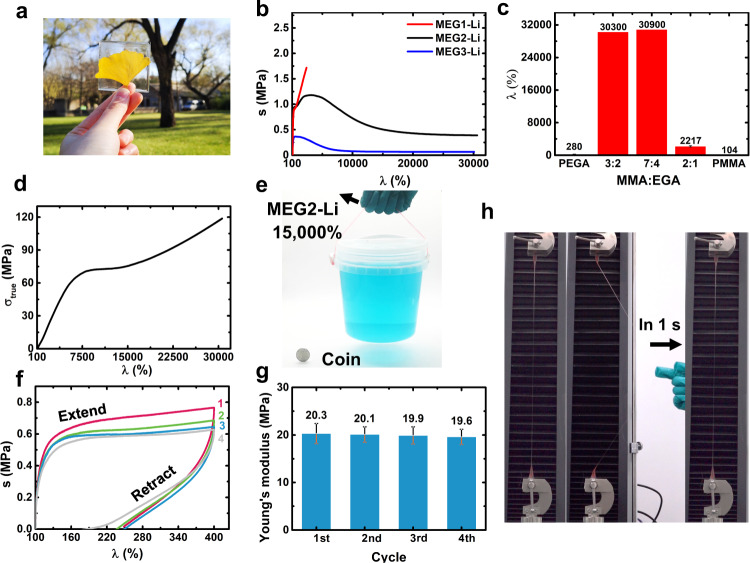
Mechanical properties. **a** The transparent MEG2-Li. **b** Tensile tests for MEG1-Li, MEG2-Li, MEG3-Li. **c** Stretch (*λ*) plotted against molar ratios of MMA/EGA. **d**, True tensile stress-strain curves of MEG2-Li. **e** The photograph of MEG2-Li lifting a 1.5 kg bucket after 15,000% stretch. **f** Stress-strain cycling tests of MEG2-Li at the rate of 10 mm/min, the interval of each cycle was 60 min. **g** The statistics of Young’s modulus for each cycle in Fig. 3f, error bars represent standard deviation. **h**, The photograph of further laterally stretching the MEG2-Li with already 30,000% stretch.

To prepare the proposed copolymers, preselected amounts of MMA monomers, EGA monomers, lithium salts, and photo-initiator were mixed. Transparent elastomers were obtained after UV radiation (Fig. 3a). To promote effective ion-dipole interactions, the molar ratio of 1:50 between lithium ions and ether oxygen atoms on EGA was determined through simulation and experimental results^33^. The ratio between MMA and EGA are 2:1, 7:4, and 3:2 for MEG1-Li, MEG2-Li, MEG3-Li, respectively. In these MEG polymers, increasing the MMA content leads to higher stiffness but lower stretchability, which indicates the higher MMA content makes for the formation of larger-size PMMA nanodomains (Fig. 3b). Surprisingly, when the ratio decreased to 7:4, the specimen could achieve superstretchability with 30,000% stretch without breaking (Supplementary Fig. 1, Supplementary Movie 1), while the polymer still exhibited a high Young’s modulus of 18 MPa (Supplementary Fig. 2). The elongations of MEG2-Li and MEG3-Li (over 30,000%) were much higher than individual PMMA (*λ* = 104%) and PEGA (*λ* = 280%) (Fig. 3c, Supplementary Table 4). Considering the shrinking of cross-section area especially over 30,000% stretch, the corrected true tensile strength of the materials better represented the actual stress, which reached as high as 110 MPa (Fig. 3d). The MEG2-Li specimen (width: 10 mm, thickness: 2.0 mm, initial length: 1.5 mm) maintained its strong mechanical strength when *λ* = 15,000%, which could lift a bucket of 1.5 kg (Supplementary Movie 2, Fig. 3e).

As shown in Fig. 3f, the stress-strain cycling curves of the materials almost overlapped with the initial state after three loadings at a stretch of 4 times, suggesting that the ion-dipole interactions could reform completely in the relaxation process (Supplementary Fig. 3). After three loading-unloading cycles, the Young’s modulus of the MEG2-Li did not change obviously (Fig. 3g). Surprisingly, the MEG2-Li polymer can remain elastic within a large range. When stretched to less than λ = 1,300%, the material could still fully return (Supplementary Fig. 4, Supplementary Fig. 5). Even at 30,000% stretch, the material remained remarkably elastic: it could be further laterally stretched and recovered within 1 s (Fig. 3h, the latter part of Supplementary Movie 1).

### High toughness and anti-fatigue performance

**Fig. 4:**
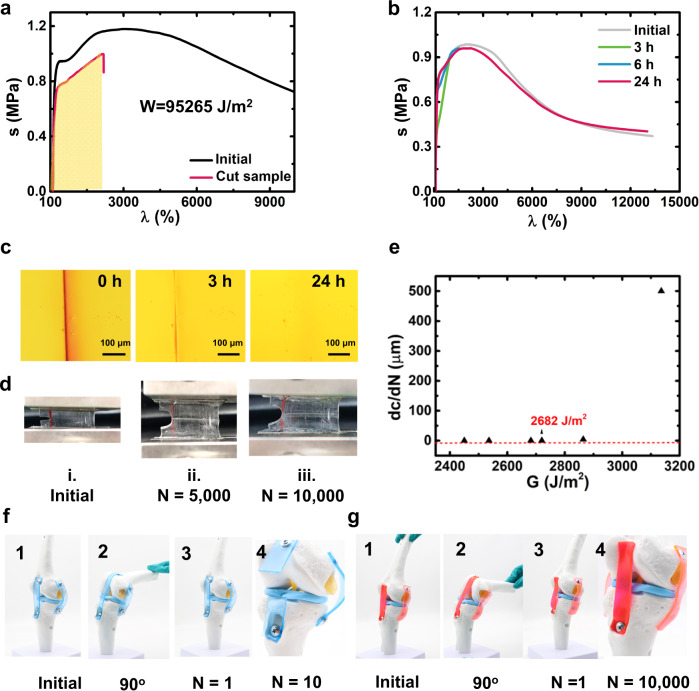
Toughness and anti-fatigue properties. **a** Fracture energy of MEG2-Li. **b** Stress-strain curves of original and healed specimens for different healing times at room temperature. **c** Optical microscope images of a cut MEG2-Li specimen healing for various times at room temperature. **d** Photos of the initial notched specimen (*N* = 1, *λ* = 100%) (i) and the notched specimen after 5,000 cycles (ii) (*N* = 5,000, *λ* = 337.5%) and after 10,000 cycles (iii) (*N*= 10,000, *λ* = 337.5%). **e** Crack propagation per loading cycle (*dc/dN*) plotted against energy release rate (*λ* = 312.5%, 322.5%, 337.5%, 350%, 362.5%, 375%). **f**, Photos of PDMS specimen as ligaments bent 90^o^ for 10 times and broken: 1. initial 2. bent 90^o^ 3. the specimen returned to the vertical state 4. the magnified pictures for the broken ligament after 10 cycles. **g** Photos of MEG2^-^Li specimen as ligaments bent 90^o^ for 10,000 times: 1. initial 2. bent 90^o^ 3. the specimen returned to the vertical state 4. the magnified pictures for intact ligaments after 10,000 cycles.

MEG2-Li also has a superior toughness as high as 228 MJ m^−3^, three orders of magnitudes higher than individual PMMA (0.11 MJ m^−3^) and PEGA (0.087 MJ m^−3^) (Supplementary Table 5). The high toughness was further visualized by an impact experiment. A 1.5 kg sharp conical hammer was released from the height of 0.1 m onto MEG2-Li sheet (thickness 1.0 mm), the polymer sheet suffered such a high impact but remained intact (Supplementary Movie 3). Figure 4a illustrates the anti-tearing ability of the materials, a 25 mm-width specimen with 5 mm notch could be stretched to *λ* = 2,100% without crack growth with a fracture energy of 95,265 J m^−2^ (Supplementary Table 6). Besides, the MEG2-Li sheet (thickness 1.0 mm) could even sustain a puncture from a sharp needle. After puncture, the elastomer quickly returned to its original state (Supplementary Fig. 6, Supplementary Movie 4). Impressively, the polymers could self-heal over time at room temperature (Supplementary Fig. 7) after cut into halves. Healing at room temperature for 24 h led to a self-healing efficiency of 98% (Fig. 4b). The self-healing process was also observable under an optical microscope (Fig. 4c).

Fatigue resistance refers to the capacity to sustain periodic stress, which is tested using cyclic pure shear tests following the procedure developed by Thomas A. G. and others^34^. The fatigue threshold is the minimum tearing energy at which mechanical rupture of single layer of chains occurs. We did fatigue tests for the crosslinked PDMS, MEG2, and MEG2-Li. The PDMS specimen broke down in one cycle when *λ* = 250% (Supplementary Fig. 8). Whereas in the tests of MEG2 and MEG2-Li elastomers, the crack did not grow even after 10,000 cycles and reached a steady state (Fig. 4d, Supplementary Fig. 9). By varying the applied stretch *λ*, we could obtain a plot of crack extension per cycle (*dc/dN*) versus the applied energy release rate (*G*). By linearly extrapolating the curve of *dc/dN* versus *G* to the intercept with the abscissa, we can approximately obtain the fatigue threshold (*Γ*_*th*_) of MEG2-Li when *λ =* 337.5% (*Γ*_*th*_ = 2,682 J m^−2^) (Fig. 4e, Supplementary Fig. 10), below which the fatigue crack will not propagate under infinite loading cycles of loads. The high fatigue threshold outperforms all the conventional rubbers and MEG polymers (Supplementary Tables 3, 7), around two orders of magnitudes higher than that of natural rubber (40 J m^−2^). The high threshold of MEG2-Li derives from the double-crosslinking structure. On the one hand, the ion-dipole interactions provide a dynamic crosslinking to eliminate the stress concentration and dissipate mechanical energy. On the other hand, the PMMA physical crosslinkers will blunt the crack^32^. Moreover, since the loosely packed PMMA nanodomains can be stretched into smaller size, it would provide additional interfacial energy to make the material resistant to multiple cycling tests, which can be validated by scaling theory discussed in the last part.

Taking advantage of the superstretchable, stiff, yet fatigue-resistant features, we further demonstrated the application of MEG2-Li as artificial ligaments for robotics. We compared the performance of crosslinked PDMS and our MEG2-Li. The experiments were carried out as following: the initial specimen was bent 90° (radius = 7.6 cm) and subsequently returned to the vertical state, the process was repeated for 10,000 times. Merely after 10 cycles, the PDMS specimen fractured completely (Fig. 4f). However, the MEG2-Li remained elastic without crack initiation even after 10,000 cycles (Fig. 4g). Besides, when our artificial ligament got injured, it could autonomously heal and sustain a bend of approximate 180^o^ and retract in less than 10 s (Supplementary Fig. 11, Supplementary Movie 5), as well as endure over 1,000 bending cycles without fracture.

### Mechanism and discussion

**Fig. 5:**
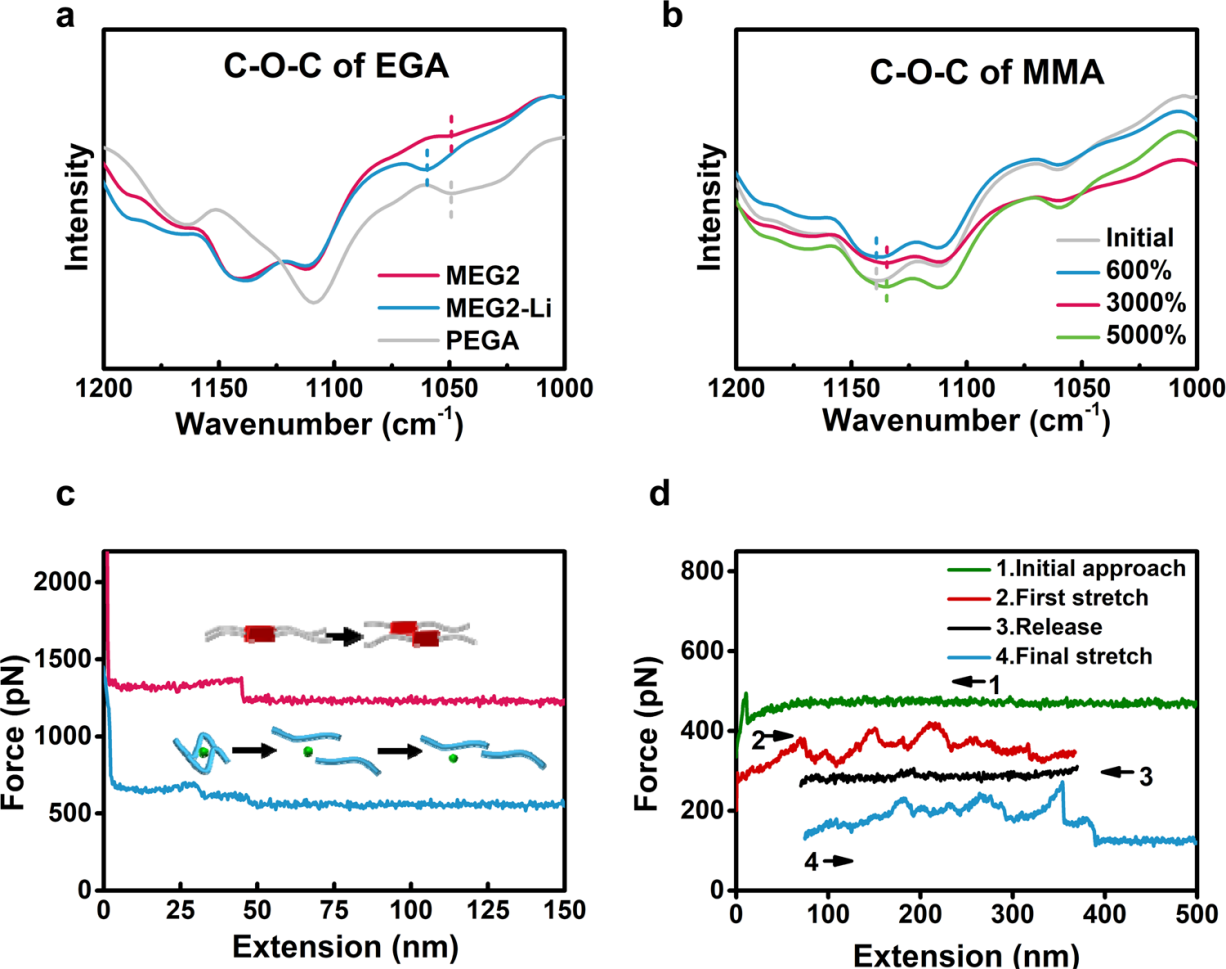
Mechanism. **a** FTIR spectrum of PEGA, MEG2, MEG2-Li. **b** The vibration changes of C-O-C on MMA side chains under stretching. **c** Typical force-extension curves during force spectroscopy measurements of the stretching of a single chain of MEG2-Li: the breakage of PMMA interactions, the breakage of Li^+^-O interactions and slippage of PEGA side chains. **d** Typical force-extension curves of MEG2-Li from stretching-releasing cycles showing reversible unfolding and refolding.

We attribute the unprecedented mechanical properties to our unique double-crosslinking design: the dynamic synergy of stiff PMMA nanoaggregates and weak ion-dipole interactions. Atomic force microscope (AFM) and fourier transform infrared spectroscopy (FTIR), Raman spectrum confirmed the double-crosslinking structure. The presence of PMMA nanoaggregates was confirmed by high-resolution AFM because of the stiffness difference between the rigid domains and amorphous parts (dark regions correspond to regions with low loss and high modulus). Pure PMMA polymer film showed an aggregate morphology (black regions). In MEG4 (MMA:EGA = 3:1), large PMMA domains (black regions) could still be observed (Supplementary Fig. 12). When the molar ratio of MMA:EGA reached 7:4, the PMMA formed amorphous nanoaggregates (Supplementary Fig. 13) which can act as dynamic physical crosslinkers. For the Li^+^-O interactions, after the addition of LiTFSI, the peak which corresponds to the asymmetric vibration of C-O-C in EGA exhibited blue shift from 1051 cm^−1^ to 1060 cm^−1^, indicating the lithium ions coordinated to the EGA side chains (Fig. 5a), in accordance with the Raman spectrum (Supplementary Fig. 14) and the Li-NMR (Supplementary Fig. 15). Moreover, the Li^+^-O bonds are more susceptible at higher frequency region (>10 Hz) which means the reformation time is less than 0.1 s (Supplementary Fig. 16). The fast reformation of Li^+^-O bonds is in favour of the elasticity of the material (Supplementary Fig. 3, Supplementary Fig. 5).

The FTIR directly proved the dynamic nature of the PMMA nanoaggregates. When we stretched the MEG2-Li within the elastic range and tested it with FTIR immediately, the peak C-O-C of the EGA did not change due to the fast reformation process, and the peak, which belongs to C-O-C of MMA did not shift either. However, when the MEG2-Li was stretched to *λ* = 3,000%, *λ* = 5000% (beyond the elastic range *λ* = 1300%), the vibration (1140 cm^−1^) exhibited obvious red shifts. This shift could be attributed to the weakened PMMA interchain interactions, indicating the uncoiling of the PMMA aggregates (Fig. 5b, Supplementary Fig. 17). To further understand the indispensable role of the dynamic PMMA nanodomains for the comprehensive mechanical properties, we developed a scaling theory of the energy changes during the stretching process. The total energy density consisted of the free energy of the PMMA/PEGA interface *F*_*int*_, and the energy of chain stretching of the middle bottlebrush PEGA block *F*_*ela*_. We analyzed the extension using the worm-like chain model^35^ when the soft-brush segments (PEGA segments) were stretched to the limit. Within strong segregation approximation^36^, the micelle morphologies of the block copolymer are obtained through minimization of free energy per block copolymer molecule. The average radius of PMMA domain (r*) is in positive correlation with the weight percentage of PMMA (*f*_*A*_) and in negative correlation with strain (*λ*). In the experiment, the radius of the PMMA domain decreases as the strain (*λ*) increases (Eq. (1), Fig. 5b). This uncoiling process provided dramatic interfacial dissipation that compensated the major energy for additional strength enhancement and anti-fatigue properties (“Supplementary Discussion 6”).

1 $${r}^{\ast }	\approx\, \frac{{C}_{2}{f}_{A}^{1/3}{\phi }^{1/3}L}{1+{C}_{1}{(\tfrac{\gamma }{{k}_{B}T})}^{-1/2}{f}_{A}^{-1/3}{\phi }^{-1/3}{p}^{-1/2}{L}^{1/2}{l}_{p}^{-1}}\\ 	 \approx \frac{{C}_{2}{f}_{A}^{1/3}{\phi }^{1/3}L{\lambda }^{-1}}{1+{C}_{1}{(\tfrac{\gamma }{{k}_{B}T})}^{-1/2}\lambda {f}_{A}^{-1/3}{\phi }^{-1/3}{L}^{1/2}{l}_{p}^{-3/2}}$$

where *C*_1_, *C*_2_ are constants.

To elucidate the origin of the superior mechanical properties at the molecular level, we performed single-molecule force spectrum (SMFS) to characterize the dynamic interchain interactions of MEG2 and MEG2-Li. Different interactions showed different peak shapes and intensities in the spectrum. The stretching of MEG2 mostly showed two kinds of interactions: strong physical interactions among PMMA nanodomains^37^ (>120 pN) and the weak Van der Walls interactions between the polymer brushes^38^ (~50 pN) (Supplementary Fig. 18, Supplementary Table 8). The MEG2-Li showed a new peak at 70–110 pN (Fig. 5c, Supplementary Fig. 18c, Supplementary Table 9), which corresponds to ion-dipole interactions between lithium ions and PEGA side chains. These results further confirmed the existence of strong PMMA crosslinkers and Li^+^-O ion-dipole interactions. To investigate whether the Li^+^-O ion-dipole interactions were highly reversible, first, we lowered the tip to contact the surface, and then stretched a chain to an extended state. We obtained a curve that indicated the successive breakage of single Li^+^-O interaction and PEGA side chains (Fig. 5d). Next, we released the unfolded MEG2-Li chain to zero force, but did not contact the surface. After waiting for 4 s, we stretched the MEG2-Li chain again to probe whether MEG2-Li chain can fold back to its initial state. The second stretching curve showed that the Li^+^-O interactions could mostly reform in a brief time and facilitate the reformation of PEGA side chains, indicating the reversible breakage and reformation of Li^+^-O interactions. In comparison, the rebinding tests of MEG2 did not yield such a force-extension curve.

Based on these results, we propose the mechanism of the stretching process as follows: when the polymer is under mechanical load, the Li^+^-O interactions will break first, then PEGA side chains begin to extend and interact with others like brushes to dissipate energy. Once the stress is removed, the Li^+^-O interactions will reform instantaneously at the newly accessible sites and render PEGA chains to return to random states due to entropic gain, thus enabling the networks to recover to the unstretched state, while the stiff PMMA crosslinkers remain intact (Supplementary Fig. 4). At larger strain, the PMMA nanoaggregates started to slide to further increase the energy-dissipating ability, thus endowing the materials with superstretchability and high strength (Fig. 5d, scaling theory).

In summary, we propose a new double-crosslinking design to achieve superstretchable, yet stiff, fatigue-resistant ligament-like elastomers. In addition to the superstretchablity and high strength brought by hierarchical energy-dissipation, the stiff PMMA nanodomains and highly dynamic Li^+^-O interactions endows the polymers with outstanding fatigue threshold. Such a design strategy can be extended to other fatigue-resistant and stiff rubbery materials which are desired in a wide range of applications in soft robotics and biomedical applications.

## Methods

### Materials

Lithium bis(trifluoromethanesulphonyl)imide (LiTFSI) was purchased from J&K Scientific, methyl methacrylate (MMA) was purchased from J&K Scientific Ltd., di(ethylene glycol) methyl ether acrylate (EGA) was purchased from TCI, and photo-initiator 2-hydroxy-2-methylpropiophenone (HMPP) was purchased from 3 A chemicals. MMA and EGA were purified with basic alumina before use. Silicone elastomer base and curing agent (SYLGARD^TM^ 184) were purchased from Dow Corning.

### MEG/MEG-Li film preparation

Purified MMA, purified EGA, photo-initiator HMPP, and LiTFSI were mixed in a 10 mL brown vial and stirred overnight to get a well-dispersed mixture. Then the liquid was poured into dumbbell-shaped Teflon molds. After polymerization for 1 h (UV light: 320 $\sim$ 400 nm, 36 W), transparent specimens were obtained. The molar ratio of MMA:EGA was 2:1, 7:4, and 3:2 for MEG1-Li, MEG2-Li and MEG3-Li, Li^+^:O = 1:50, where O is referred to the ether oxygen atoms. MEG2 (MMA:EGA = 7:4, without lithium salts), MEG4 (MMA:EGA = 3:1, without lithium salts). The content of the photo-initiator was about 80 μL per 6 mL monomer solution.

### PDMS film preparation

The mixture of silicone elastomer base (10 g) and silicone elastomer curing agent (1 g) were stirred well in a beaker and poured into the flat culture dish. After the removal of air bubbles, the mixture was then cured at 80 °C for 3 h in oven. Finally, PDMS films were cut into rectangular shape.

### Mechanical test

Mechanical tests were performed using Sunstest UTM2502. Unless otherwise noted, all tensile tests were performed at room temperature (24 ± 1 °C) at the loading rate of 10 mm min^−1^ (6.7 min^−1^), the size of the specimens for tensile tests is 6 mm × 1.8 ± 0.1 mm (W × T).

The Young’s modulus is measured by initial slope of the stress-strain curve. Toughness is defined as an integral area under stress-strain curve.

The healing tests were performed by gently putting the two cut pieces together. To observe the incision well, the healed specimen was clamped by two grips with a 4 mm gap in the initial state. All the results were repeated for over 3 times using different batches of specimens. The healing efficiency is defined as the proportion of toughness restored relative to the original toughness.

For cyclic stress-strain tests, the stretching and releasing rate was 10 mm min^−1^, the relaxation time was 45 min or 60 min.

For fracture energy tests, pure shear tests were performed at a stretching rate of 10 mm min^−1^, the quadrate specimens are 30 mm wide, 2 mm thick, with a gauge length of 4.5 mm and a crack width of 5 mm. The critical stretch (*ε*_*c*_) for the notched specimens was obtained from the stretch at the break. The pairing unnotched specimens were stretched until *λ = ε*_c_. The fracture energy value (*F*) was obtained by multiplying the area under the stress-strain curve of the unnotched specimens with the initial clamp distance, *F* = HW(ε_*c*_), W(ε_*c*_) is the elastic strain energy density of the unnotched specimen when *λ = ε*_*c*_, *H* is gauge length.

### Fatigue test

To verify the fatigue resistance of the MEG polymers, pure shear tests were performed, which were widely used in fatigue tests of rubbers. All the fatigue tests were performed in air at 24 ± 1 °C using Sunstest UTM2502, the cycling tensile rate was 50 mm min^−1^ (12.5 mm^−1^). All the quadrate specimens were 30 mm wide, 2 mm thick, with a gauge length of 4 mm and a crack width of 5 mm (the crack length should be larger than the gauge length). A digital camera (C920e, Logitech) was used to monitor the crack propagation of the materials and screenshot software was used to record the crack extension.

For pure shear tests, two identical specimens (one notched, one unnotched) were loaded under the specimen setup as a pair. The notched specimens were used to obtain the *λ* and the stress-strain curves of the unnotched specimens were used to calculate the energy release rate value *G*.

$$G={HW}({\lambda }),{\varGamma }_{{th}}={HW}({\lambda }c)$$

*W*(*λ*) is the elastic strain energy density of the unnotched specimen, *H* is the gauge length. The critical stretch *λ*_*c*_ is the maximum *λ* when the crack of the notched specimen does not grow even after thousands of cycles. When *λ*=*λ*_*c*_, the energy release rate *G* is the fatigue threshold *Γ*_*th*_. The fatigue threshold is calculated when the stress-strain curve of the unnotched specimen reach a steady state.

### Single-molecule force spectrum

Single-molecule force experiments on MEG2 and MEG2-Li were performed on a JPK Nanowizard IV, each Si_3_N_4_ AFM cantilever (MLCT D type, Bruker, Santa Barbara, CA) was calibrated in water before each experiment, showing a spring constant of around 48 pN nm^−1^. All experiments were performed in water at room temperature. For the experiment of MEG2 and MEG2-Li, toluene solution (0.5 mg mL^−1^) was used. In a typical experiment, the solution was deposited on a clean glass coverslip and allowed to dry. One drop of water was added before stretching. The macromolecules were then stretched under a constant pulling speed of 1000 nm s^−1^. To investigate whether the unfolding and folding of MEG2-Li was reversible, we released the unfolded MEG2-Li chain quickly to zero force. After waiting for 4 s, we stretched the MEG2/MEG2-Li again to probe whether it could fold back to its original state. In order to perform single-molecule force measurements, the molecules need to be at low density. Therefore, in single-molecule force experiments, the events mainly correspond to the rupture of intramolecular interactions. In contrast, in MEG2-Li elastomers, both intramolecular and intermolecular interactions are broken upon stretching. Despite of their different rupture modes, the chemical nature of the interactions is the same.

Optical micrographs were recorded with a cross-polarized optical microscope (Nikon LV100N POL). FTIR data were recorded on a Horiba Bruker FTIR. Raman data were recorded on a LabRAM HR Evolution. AFM images were recorded on an Asylum Research Cypher ES. The dark regions with low phase angle correspond to regions with relatively low loss and high modulus, and the bright regions with high phase angle correspond to regions with relatively high loss and high modulus.

DSC measurements were performed using a TA Instruments DSC 250 with a heating speed of 5 °C min^−1^. DMA measurements were performed using TA Instruments DMA 850 from 0.1 Hz to 100 Hz at about 25 °C.

NMR spectra were acquired on a JEOL JNM-ECZ400S spectrometer with a HX probe (operating at 155 MHz for 7 Li), equipped with a z-gradient coil producing a nominal maximum gradient of 0.3 T m^−1^. Specimens containing 0.064 mmol LiTFSI and 1 mmol EGA monomer (Li^+^:O = 1:50) in a 5 mm NMR tube were evaluated. For the Li diffusion measurements (DOSY), the experiments were carried out applying a stimulated-echo NMR pulse sequence with the delay for gradient recovery and duration of the gradient purge pulse at 6 and 150 ms respectively. Sixteen nominal gradient amplitudes ranging from 0.02 to 0.3 T m^−1^ were chosen to give equal steps in gradient squared; each FID was acquired using 32k data points.

## Supplementary information

Supplementary Information

Description of Additional Supplementary Files

Supplementary Movie 1

Supplementary Movie 2

Supplementary Movie 3

Supplementary Movie 4

Supplementary Movie 5

## Data Availability

The authors declare that the main data supporting the findings of this study are available within the paper and its Supplementary Information files.
