# Supplementary material for: Superstretchable, yet stiff, fatigue-resistant ligament-like elastomers
Source: Nat Commun. 2022 Apr 27;13:2279. doi: 10.1038/s41467-022-30021-3 (PMC9046184; doi:10.1038/s41467-022-30021-3)
Supplement: Supplementary file 1 — Supplementary Information [file 41467_2022_30021_MOESM1_ESM.pdf]

Supplementary Information for

Superstretchable, yet stiff, fatigue-resistant ligament-like elastomers

Mengxue Li[1†], Lili Chen[1†], Xiaobin Dai[3] Yiran Li[2], Zhekai Jin[1], Yucheng Zhang[1], Wenwen Feng[1], Li-Tang Yan[3], Yi Cao[2], Chao Wang[1]*

Correspondence to: chaowangthu@mail.tsinghua.edu.cn

This PDF file includes:

Supplementary Discussions 1 to 7
Supplementary Figures 1 to 18
Supplementary Tables 1 to 9
Supplementary References 1 to 10

Supplementary Discussions

Supplementary Discussion 1: Contradiction between fatigue threshold and stiffness

Stiffness shows the resistance to deformation and is measured by the Young's modulus. The relation between stiffness (E) and Young's modulus (Y): $E = YL_0/S$. S is the initial cross-sectional area of the specimen, L_0 is the initial effective length of the specimen.

Fatigue threshold measures the maximal capacity to periodic stress. A single-network elastomer is composed of crosslinked network of flexible polymer chains. Stiffness is estimated by $E=3kT/(Vnl)$, where V is the volume per monomer, n is the number of monomers per polymer chain, l is the length of the monomer unit, kT is the temperature in the unit of energy[1]. The fatigue threshold is estimated by the Lake-Thomas model, $\Gamma_{th} = \alpha\sqrt{n}\ lJ/V$, where α is a dimensionless number of order unity, J is the energy per covalent bond[2].

Because of the intrinsic conflict from a fundamental fact: the stiffness $E{\sim}1/n$, the threshold $\Gamma_{th}{\sim}\sqrt{n}$, So the stiffness E decreases with the number of monomers per polymer chain n increasing, while the threshold Γ_{th} increases with the increasing of n. Consequently, it is hard to achieve both high stiffness and high threshold in a single network elastomer. Various elastomers have been measured to confirm the stiffness-threshold conflict [3].

Supplementary Discussion 2: Contradiction between stiffness and stretchability

According to the flexible chain theory in polymer physics, the ratio between mean square end distance of completely extended polymer chains and freely rotating polymer chains determines the maximum tensile ratio λ_{max} of polymer. The mean square end distance of completely extended polymer chains is $\sqrt{2/3}Nl$ (N is the number of bonds in the main chain, l is the length of bond); And the mean square end distance of freely rotating polymer chains is $\sqrt{2Nl}$. Thus, the maximum tensile ratio $\lambda_{max}=\sqrt{N/3}$. And the number of bonds N is proportional to the the number of monomers per polymer chain n, so $\lambda_{max}{\sim}\sqrt{N}{\sim}\sqrt{n}$.

Due to the stiffness $E{\sim}1/n$, the maximum tensile ratio $\lambda_{max}{\sim}\sqrt{n}$, the stiffness E and the maximum tensile ratio λ_{max} change reversely as n increases. Thus, high-modulus polymer materials usually accompany with low stretchability.

Supplementary Discussion 3: The advantage of dynamic double crosslinking design

In our double crosslinking design, the polymer network is connected by two crosslinkers which differ greatly in strength. The rigid PMMA crosslinkers mainly provide enough stiffness to resist the high external pressure. Whereas at high stretch, besides the energy-dissipating ability of the Li^{+}-O bonds and PEGA brushes, PMMA

nanodomains can uncoil to improve the stretchability and strength. The double crosslinking design makes for the combination of high modulus and high elongation in a single polymer network.

As for the fatigue threshold, previous researches claimed that the delocalization of the stress concentration and strong hard parts (eg. crystal regions[4] or stiff fibers[5]) for resisting cracks are important to improve the fatigue threshold. Recently, Gong et al have proposed that physical crosslinkers can also effectively prevent the chain pullout [6]. In the MEG2-Li, the soft PEGA blocks can act as transfer media to delocalize the stress to distal PMMA blocks, and the weak Li^+-O crosslinkers can break preferentially and could reform reversibly to dissipate energy repeatedly. Moreover, the stiff and PMMA crosslinkers ahead the crack will restrict the chain pullout and blunt the crack. And after multiple cycles, PMMA nanodomains could further uncoil to provide additional interfacial energy to resist the crack formation.

Supplementary Discussion 4: The explanation for the high fracture energy

To investigate the mechanism of the anti-tearing properties for MEG2-Li, we further tested the fracture energy of MEG1-Li and MEG3-Li. The fracture energy of MEG2-Li is the highest compared with MEG1-Li and MEG3-Li (Supplementary Table 6). Thus, we infer that the anti-tearing ability derives from the synergy of the stiff PMMA nanodomains and the highly dissipative amorphous parts in MEG2-Li elastomer. When the notched sample is stretched, the portion close to the notch is stretched more than elsewhere. In the first stage, the weak Li^+-O bonds break first and PEG brushes slip to delocalize the energy with intact stiff PMMA nanodomains to resist crack growth. In the second stage, the PMMA nanodomains can uncoil to further dissipate energy until the materials break. The explanation of the anti-tearing ability is analogous to the mechanism of anti-fatigue properties for MEG polymers, and the difference between these two properties remains a challenge and needs further research in the mechanics area.

Supplementary Discussion 5: The definition of fatigue threshold

The loading-unloading hysteresis loop is decreased after thousands of cycles due to the segmental motion and partial reformation of Li^+-O interactions. After about successive 10,000 cycles, the steady stress-strain curve can be obtained. But after 12 hours, the MEG-Li can return to the 60% of its initial stress. Even after another 10,000 cycles and relaxation time of 12 hours, the property can still recover (Fig. S6). The anti-fatigue mechanism of the MEG2-Li is different from the conventional covalently-bonded networks which comply with the classical Lake-Thomas theory. When the material was placed for 12 h, most of the Li^+-O interactions in the material could be restored. Here, we choose the tensile curve of the material after recovery for 12 h to calculate the fatigue threshold. The synergy of physical crosslinking and strong

dissipative ability can help continuously recover the anti-fatigue properties of the material, thus greatly improving the service life of the material.

Supplementary Discussion 6: Theoretical calculations

To understand the role of the dynamic PMMA nanodomains and the comprehensive mechanical properties, we develop a scaling theory for the self-assembly of $(AB)_n$ block copolymers. Detailed calculations of the free energies of domains in a micelle formed by copolymers with arbitrary branched architecture of blocks are presented as follows.

Within strong segregation approximation[7], the micelle morphologies of block copolymer are obtained through minimization of free energy per block copolymer molecule,

$$F_{tot} = F_{ela} + F_{int} \quad (1)$$

which is composed of contribution of two parts: a. the free energy of the A/B interface F_{int}, and b. the energy of chain stretching of the middle bottlebrush B block F_{ela}. The contribution from the stretching of the end blocks is neglected as they are much smaller than that of the middle block as discussed in the end.

Let us consider a spherical micelle self-assembled by a diblock copolymer comprising an end block A with a degree of polymerization N_A, and bottlebrush block B with a degree of polymerization N_B. The weight fraction of end blocks is f_A, the average radius of a sphere domain is R, and the distance between the centers of two neighboring spherical domains is λd_0, where d_0 is the initial length, and λ is the stretch ratio. In the case of the uniaxial deformation at a constant volume the elastomer extends in one direction along the x-axis $\lambda_x = \lambda$ while it contracts in two others $\lambda_y = \lambda_z = \lambda^{-1/2}$.

Within a volume V and the packing fraction of the block copolymer ϕ, there are $n_s \approx V/(d_0^3\phi)$ spherical domains. The total interfacial area is $A \approx 4\pi n_s r^2$. Considering that the density of different blocks is nearly the same,

$$f_A = \frac{V_A}{V} \approx \frac{\frac{4}{3}\pi r^3 n_s}{n_s d_0^3 \phi} = \left(\frac{4\pi}{3\phi}\right)\frac{r^3}{d_0^3} \quad (2)$$

Therefore, the interfacial area is

$$A = \frac{3\phi V f_A}{r} \quad (3)$$

The interfacial energy density is the product of interfacial tension γ and the interfacial area normalized by volume,

$$F_{int} = \frac{\gamma A}{V} = \frac{3\phi f_A \gamma}{r} \quad (4)$$

which increases linearly with f_A but is inverse to the average radius of spherical domains.

The stretching free energy of the middle block treated as a worm-like chain in strong limits can be analyzed below[8].

For a worm-like chain, the average radius gyration of B block $\left\langle R_{0,B}^2 \right\rangle$ relates to its persistence length, l_p, and the contour length L,

$$\left\langle R_{0,B}^2 \right\rangle = 2l_p L \quad (5)$$

and the chain volume can be written as

$$V_c = p\left\langle R_{0,B}^2 \right\rangle = 2pl_p L \quad (6)$$

where p is the packing length of the bottlebrush block.

For a bottlebrush, the effective Kuhn monomer is nearly spherical and thus the packing length is comparable to the persistence length, $p \approx l_p$.

In the strong stretching limit when the polymers reach a superstretched state, the end-to-end distance of the B block approaches the contour length L at large force. The force-extension rule of the worm-like chain near the full extension is given by

$$\frac{\sigma l_p}{k_B T} = \frac{1}{4}(1-\frac{z}{L})^{-2} - \frac{1}{4} + \frac{z}{L} \qquad (7)$$

where σ is the force applied to the ends of B block at separation z. Integrating this equation by starting from an arbitrary reference $R_{z,0}$ gives the free energy,

$$\begin{aligned} F(d) &= -W = -\int_{R_{z,0}}^{d} \sigma dz = -\frac{k_B TL}{l_p}\left(\frac{1}{4}(1-\frac{z}{L})^{-1} - \frac{1}{4}\frac{z}{L} + \frac{1}{2}(\frac{z}{L})^2\right)\Bigg|_{R_{z,0}}^{\lambda d_0} \\ &= \frac{k_B TL^2}{4l_p(L-d)} - \frac{1}{4}\frac{\lambda d_0}{L} + \frac{1}{2}(\frac{\lambda d_0}{L})^2 + const \approx \frac{k_B TL^2}{4l_p(L-\lambda d_0)} + const \end{aligned} \qquad (8)$$

where the divergence at the $d = L$ restricts the full extension. The total free energy density in this limit is given by

$$F_{tot} \approx F_{ela} + F_{int} = \frac{1}{V_c}\frac{k_B TL^2}{4l_p(L-\lambda d_0)} + \frac{3\phi\gamma f_A}{r} = \frac{k_B TL^2}{8pLl_p^2(L-\lambda r(\frac{4\pi}{3\phi f_A})^{1/3})} + \frac{3\phi\gamma f_A}{r} \quad (9)$$

Minimizing the free energy by varying r yields,

$$r^* \approx \frac{C_2 f_A^{1/3}\phi^{1/3}L}{1+C_1(\frac{\gamma}{k_B T})^{-1/2} f_A^{-1/3}\phi^{-1/3}p^{-1/2}L^{1/2}l_p^{-1}} \approx \frac{C_2 f_A^{1/3}\phi^{1/3}L\lambda^{-1}}{1+C_1(\frac{\gamma}{k_B T})^{-1/2}\lambda f_A^{-1/3}\phi^{-1/3}L^{1/2}l_p^{-3/2}} \qquad (10)$$

where C_1, C_2 are constants.

Molecular parameters of bottlebrush polymers. The contour length L, persistent length l_p, and end-to-end distance R of bottlebrush block are calculated based on its molecular architecture[9]. Consider a densely grafted bottlebrush block B has n_{sc} side chains, N_{sc} Kuhn monomers, and N_{Li}, the number of lithium ion on each side chain. The contour length is proportional to number of side chains

$$L = n_{sc} l \quad (11)$$

in which l is the distance between two neighboring grafting sites along the backbone. The persistence length, l_p, of a densely grafted bottlebrush is about its cross-section size, which was described by others. For a bottlebrush block, the side chains are densely grafted to a backbone polymer, occupying a cylindrical space surrounding the backbone.

The cross-section size of the cylindrical space is about the average radius R_{sc} of a side chain. Within such a cylindrical space, a side chain occupies a volume, $R_{sc}^2 l$, that is the product of the cross-section area R_{sc} and the distance between two neighboring grafting sites l. This volume is equal to the volume of a side chain in total, $N_{sc}v_0 + N_{Li}v_{Li}$, in which v_0 is the volume of a Kuhn monomer of bottlebrush, and v_{Li} is the volume of a lithium ion. Therefore, the cross-section size of the bottlebrush is $R_{sc} \approx \left(\frac{N_{sc}v_0 + N_{Li}v_{Li}}{l}\right)^{1/2}$. The persistence length of the bottlebrush polymer is about its cross-section size,

$$l_p \approx R_{sc} \approx \left(\frac{N_{sc}v_0 + N_{Li}v_{Li}}{l}\right)^{1/2} \quad (12)$$

The effect of stretching ratio. For a broad class of unentangled polymer networks, it has been demonstrated[8, 10] that the stress-strain relation can be expressed in universal form as:

$$\sigma_{true} = \frac{G}{3}(\lambda^2 - \lambda^{-1})\left[1 + 2\left(1 - \frac{\beta(\lambda^2 + 2/\lambda)}{3}\right)^{-2}\right] \quad (13)$$

where σ_{true} is the true stress of polymer elastomer, G is the modulus of rigidity, and $\beta = \left\langle R_0^2 \right\rangle / R_{max}^2$ is the ratio of the mean-square average end-to-end distance of the strand in the undeformed network to the square of the end-to-end distance of the fully extended strand R_{max}^2. In the worm-like chain model, $\beta = \frac{2l_p}{L}$.

The shape of PMMA domains

If the domain with radius r deforms at constant volume V, they will become a prolate spheroid like a symmetrical egg with the polar radius c and equatorial radius a(c>a). We present the expressions of the volume and surface area of a sphere and a prolate spheroid,

$$V = \frac{4}{3}\pi r^3 = \frac{4}{3}\pi a^2 c \quad (14)$$

$$S = 2\pi a^2 + 2\pi \frac{ac}{\sqrt{c^2 - a^2}} \arcsin\left(\frac{\sqrt{c^2 - a^2}}{c} \right) \qquad (15)$$

By substituting Equation (15) into Equation (16), we get

$$S = 2\pi \frac{r^3}{c} + 2\pi \left(\frac{r^3}{c} \right)^{1/2} \frac{\arcsin\left(\sqrt{1 - \frac{r^3}{c^3}} \right)}{\sqrt{1 - \frac{r^3}{c^3}}} \qquad (16)$$

To evaluate the maximized surface area, we need to obtain the differential:

$$\frac{\partial S}{\partial c} = -2\pi \frac{r^3}{c^2} \left[1 + \frac{\arcsin\left(\sqrt{1 - \frac{r^3}{c^3}} \right)}{2\left(\frac{r^3}{c} \right)^{1/2} \sqrt{1 - \frac{r^3}{c^3}}} + \frac{3\arcsin\left(\sqrt{1 - \frac{r^3}{c^3}} \right)\left(\frac{r^3}{c} \right)^{1/2}}{2c^2 \sqrt{1 - \frac{r^3}{c^3}}} + \frac{3\left(\frac{r^3}{c} \right)^{1/2}}{2c\sqrt{1 - \frac{r^3}{c^3}}} \right] < 0$$

(17)

Equation (17) shows that as the polar radius c increases, the surface area decreases. Therefore, the domain transforms into a sphere(c=a) to maximize the surface area at constant volume.

Supplementary Discussion 7: The influence of temperature on PMMA domains.

1) From the scaling theory, the total free energy of the system is the comprehensive result of the inverse interactions brought by the elastic energy and interfacial energy (equation (1)). As the temperature increases, the elastic energy term (F_{int}/k_BT) keeps constant while the interfacial energy term (F_{int}/k_BT) tends to decrease.

$$\frac{F_{tot}}{k_BT} \approx \frac{F_{ela}}{k_BT} + \frac{F_{int}}{k_BT} = \frac{1}{V_c} \frac{L^2}{4l_p(L - \lambda d_0)} + \frac{3\phi\gamma f_A}{k_BT}\frac{1}{r} = \frac{L^2}{8pLl_p^2(L - \lambda r(\frac{4\pi}{3\phi f_A})^{1/3})} + \frac{3\phi\gamma f_A}{k_BT}\frac{1}{r}$$

(18)

To compensate for the loss of interfacial energy, the domain size r^* decrease when the system reaches thermodynamic equilibrium (equation (2)).

$$r^* \approx \frac{C_2 f_A^{1/3} \phi^{1/3} L \lambda^{-1}}{1 + C_1 (\frac{\gamma}{k_BT})^{-1/2} \lambda f_A^{-1/3} \phi^{-1/3} L^{1/2} l_p^{-3/2}} \qquad (10)$$

2) PMMA nanodomains as physical crosslinkers mainly contribute to the elastic modulus of the materials. Larger PMMA domains are more rigid and provide stronger

crosslinking effect, leading to higher elastic modulus. Therefore, the elastic modulus values have positive correlation with the size of the PMMA domains. As shown in Supplementary Figure 16b, the elastic modulus decreases as the temperature increase, indicating that the PMMA domain sizes indeed become smaller as temperatures increase.

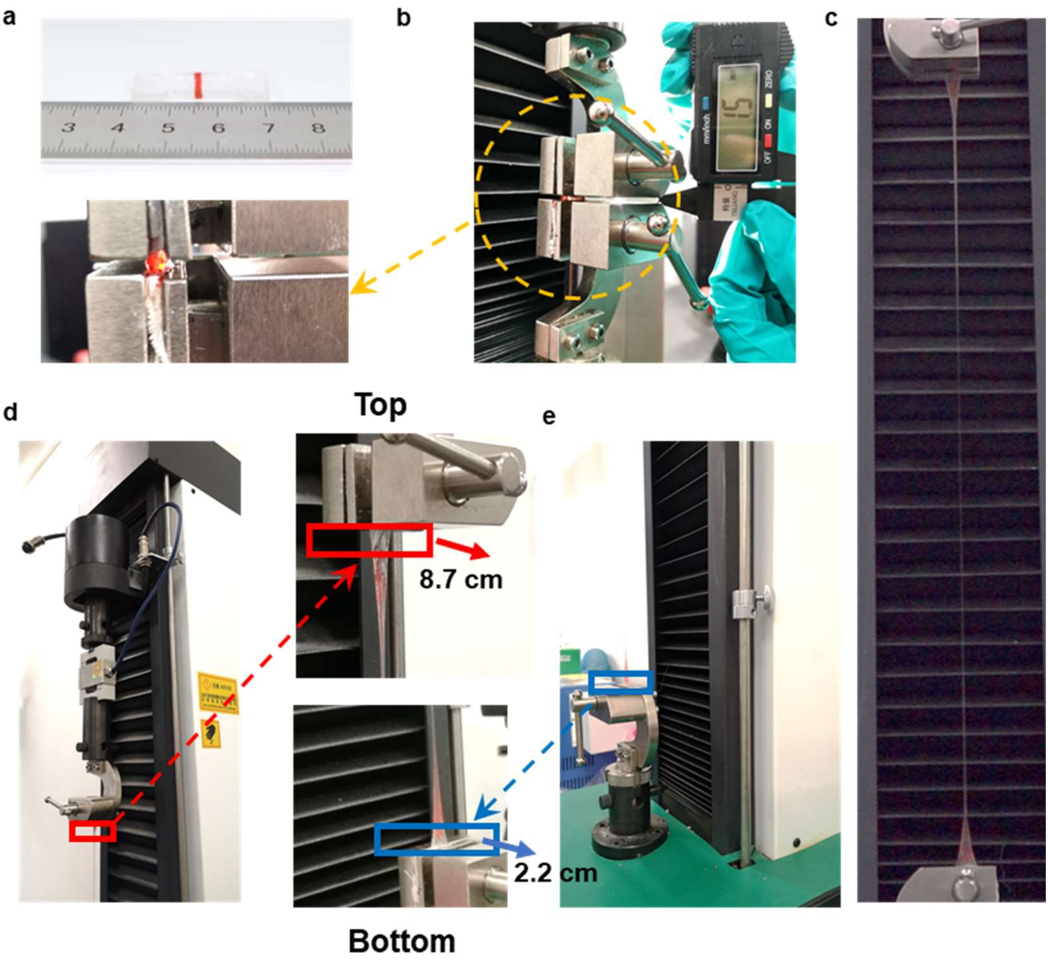

Supplementary Figure 1.

Details of tensile tests. a, The middle 1.5 mm-long specimen between the grips was painted red. **b,** The red part (1.5 mm) of the specimen was clamped between two grips after adjusting the initial force to zero. **c,** The red part (1.5 mm) of the specimen was stretched to 450 mm (~300 times as initial length, the image contrast is reduced to make the thin material visible). **d,** The top of the elongated specimen (~300 times) slipped out of the clamp for 8.7 mm (transparent part). **e,** The bottom of the elongated specimen (~300 times) slipped out of the clamp for 2.2 mm (transparent part).

It is indeed difficult to measure 30,000% strain in a stress-strain test with a standard specimen and tensile tester. Herein, we have to choose a super-short gap of 1.5 mm in our testing. To show the super-stretch process, in Supplementary Movie 1, a specimen of 20 mm $\times$ 10 mm $\times$ 1.8 $\pm$ 0.1 mm was clamped between two grips with a gap of 1.5 mm, the specimen in the gap was dyed red (The red dye is physically fixed to the specimen, Figure. S1A). The 1.5 mm gap was confirmed again with a Vernier caliper (Figure. S1B). A camera was set up to monitor and capture the stretching process. Over the entire testing length, the slippage was limited. At 30,000% stretch (Figure. S1C,

450 mm), the undyed parts were 8.7 mm (top) and 2.2 mm (bottom), only taking up 2.4% of the whole tensile stretch, as shown in Figure. S1D and Figure. S1E.

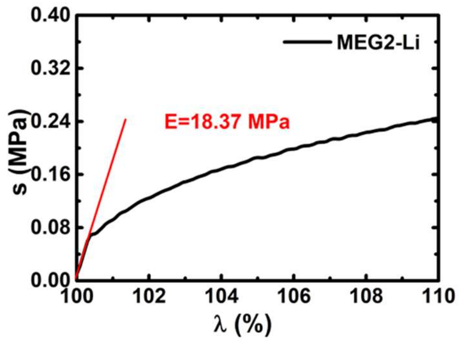

Supplementary Figure 2.

Magnified picture of stress-strain curve for MEG2-Li (MMA:EGA=7:4, Li^{+}:O=1:50), which was used to calculate the Young's modulus.

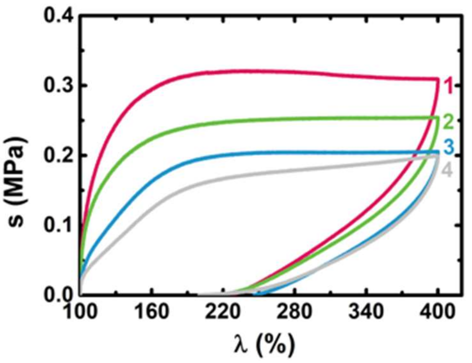

Supplementary Figure 3.

Stress-strain cycling tests. Cycling tests of MEG2 (MMA:EGA=7:4, without lithium salts), the loading rate is 10 mm/min, the interval of each cycle was 60 min.

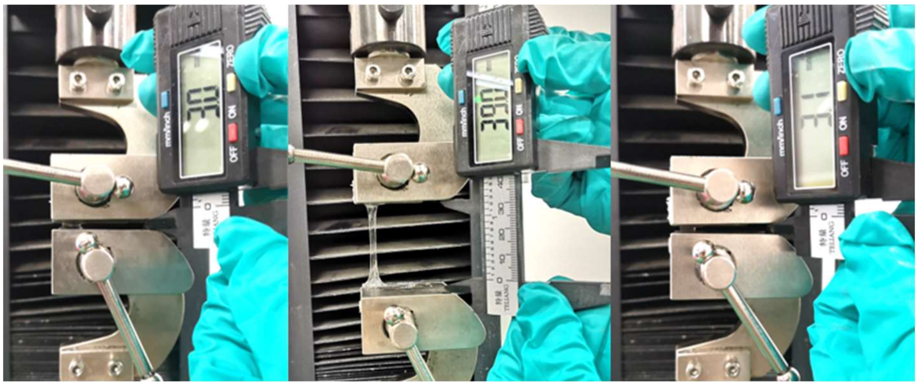

Supplementary Figure 4.

The relaxation process of MEG2-Li from λ=1,300% in 12 h.

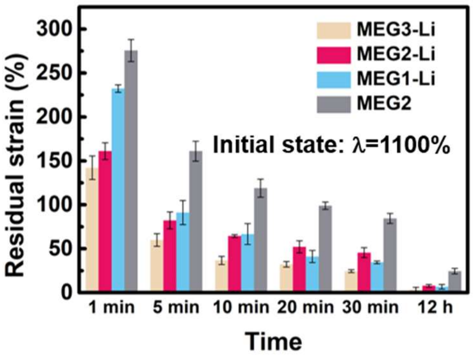

Supplementary Figure 5.

Residual strain statistic for MEG1-Li, MEG2-Li, MEG3-Li and MEG2 under λ=1,100% for different relaxation time, error bars represent standard deviation.

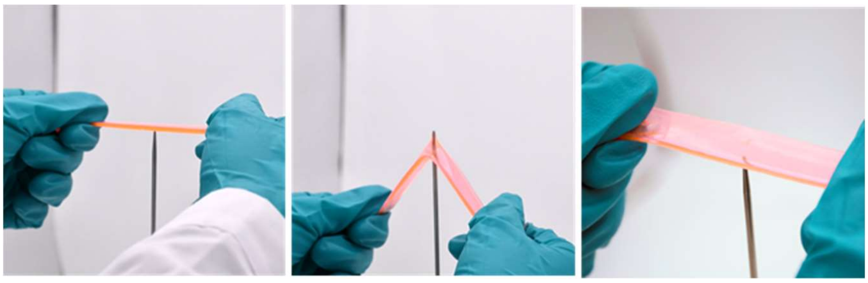

Supplementary Figure 6.

Puncture test of MEG2-Li. The film thickness: 1.8 mm.

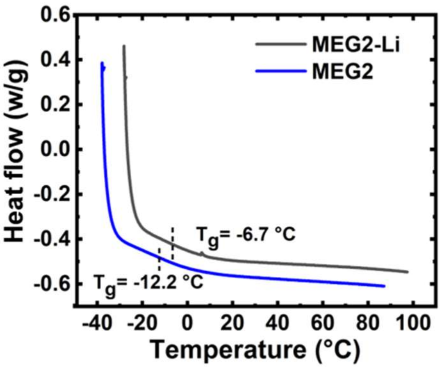

Supplementary Figure 7.

DSC data for MEG2-Li and MEG2 with a heating speed of 5 °C/min.

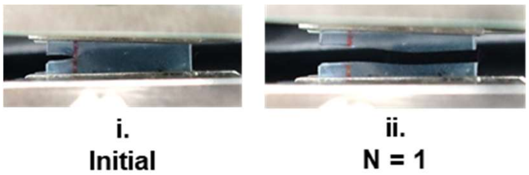

Supplementary Figure 8.

Photos of the initial notched PDMS (i) and the notched specimen after 1 cycle (N = 1, λ= 150%) (ii).

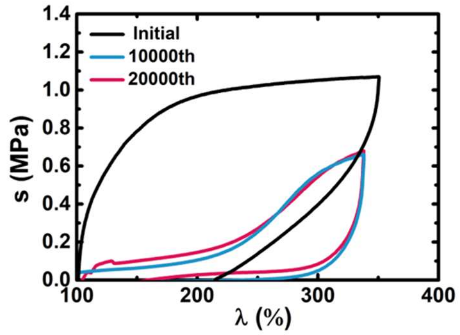

Supplementary Figure 9.

The mechanical properties of MEG2-Li reached a steady state when N=10,000 (the stress-strain cycling curve of the 10,000$_{th}$ almost overlapped with that of the 20,000$_{th}$) with the relaxation time of 12 h. When the material was placed for 12 h, most of the Li^{+}-O interactions could be restored, thus the mechanical properties recovered partly.

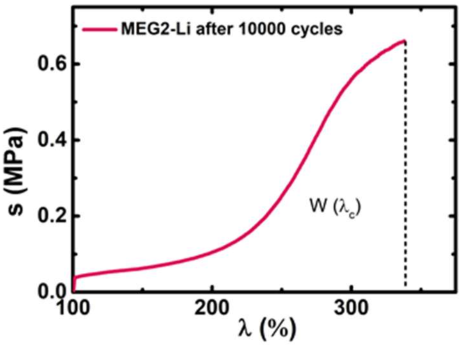

Supplementary Figure 10.

The stress-strain curve of unnotched MEG2-Li specimen after 10,000 cycles, the $W(\lambda_c)$ was the elastic strain energy density of MEG2-Li when $\lambda = 337.5\%$.

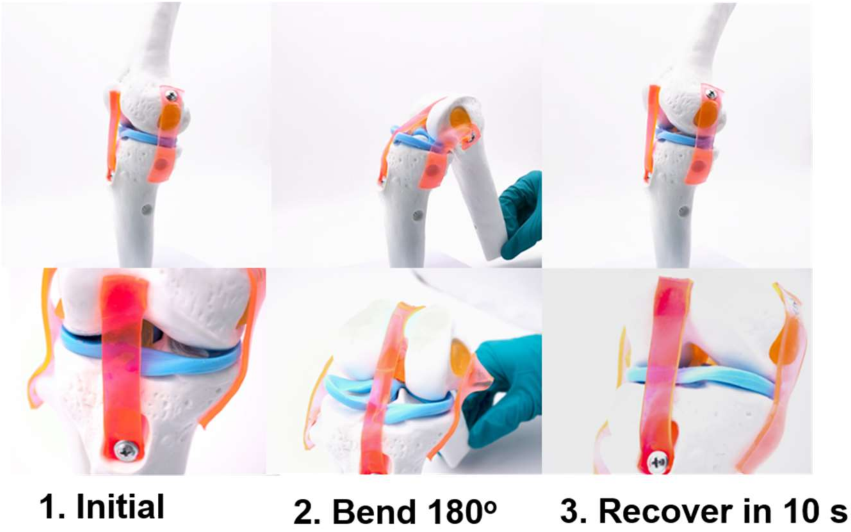

Supplementary Figure 11.

The completely healed MEG2-Li (MMA:EGA=7:4, Li^+:O=1:50) specimen was used as a ligament and bent 180°. Impressively, the specimen could almost recover in less than 10 s, showing excellent elasticity at large scale even after healing.

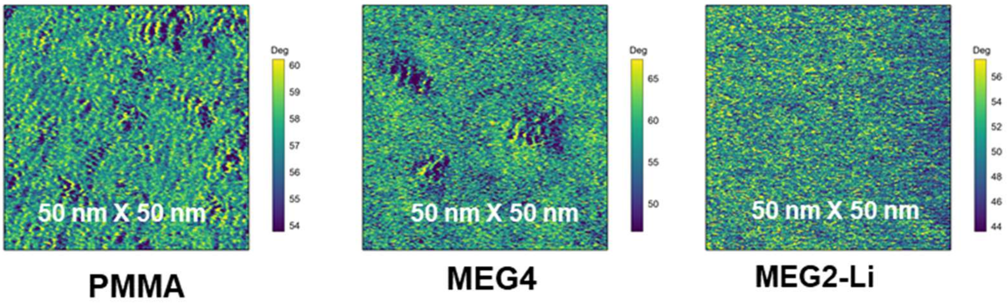

Supplementary Figure 12.

AFM phase images of PMMA, MEG4 (MMA:EGA=3:1, without lithium salts) and MEG2-Li (MMA:EGA=7:4, Li^+:O=1:50) specimens.

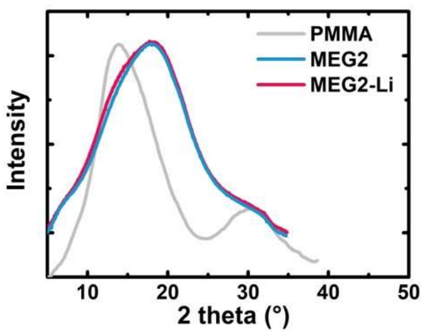

Supplementary Figure 13.

XRD data for PMMA, MEG2 and MEG2-Li. The broad diffraction peak indicates the low crystallinity of PMMA, and the crystallinities of MEG polymers are even lower, which means all these materials are at amorphous states.

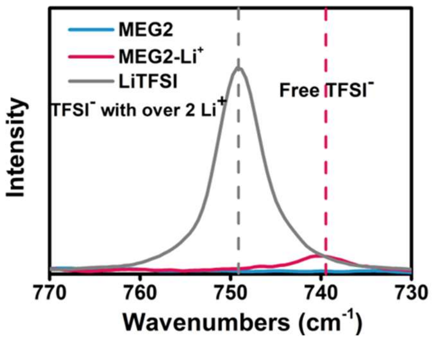

Supplementary Figure 14.

Raman spectrum of LiTFSI, MEG2, MEG2-Li. The peak of $TFSI^-$ counterions corresponding to oxygen environment (≈749 cm^{-1}) was stronger than the free $TFSI^-$ (≈742 cm^{-1}) counter ions in MEG2-Li, suggesting that most of the lithium ions were coordinated via the ion-dipole interactions.

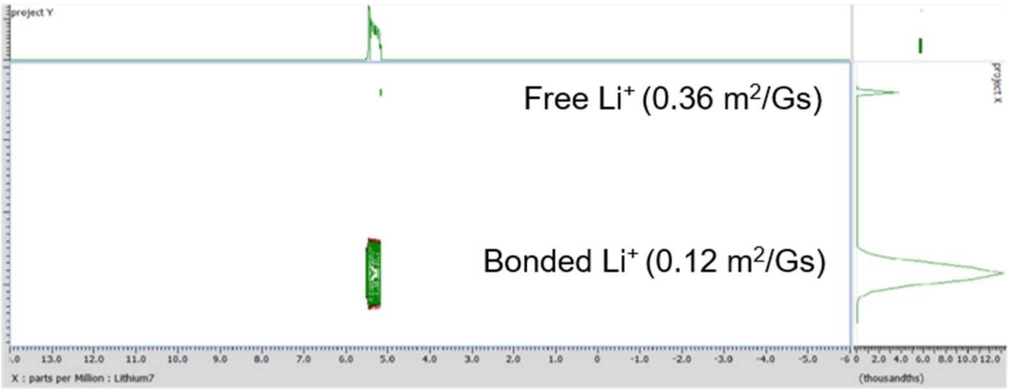

Supplementary Figure 15.

The diffusion measurements of Li^+ in liquid EGA monomer (Li^+:O=1:50). Li NMR of LiTFSI-EGA (Li^+-O) at 25°C mainly showed two kinds of state of Li^+ which corresponded to the free Li^+ and Li^+ bonded to the EGA side chains respectively. These results confirmed that the Li ions were able to interact with ether oxygen atoms on the EGA side chains.

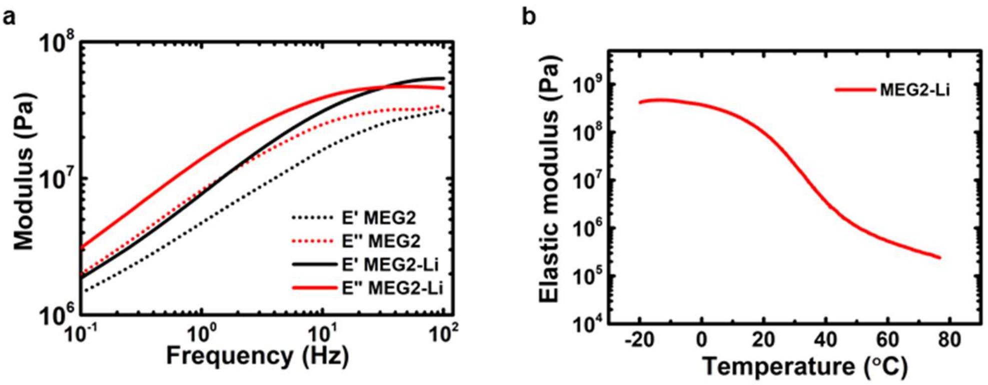

Supplementary Figure16.

a, DMA data for MEG2 and MEG2-Li from 0.1 Hz to 100 Hz at room temperature (~25 °C). **b,** The elastic modulus of MEG2-Li at different temperatures.

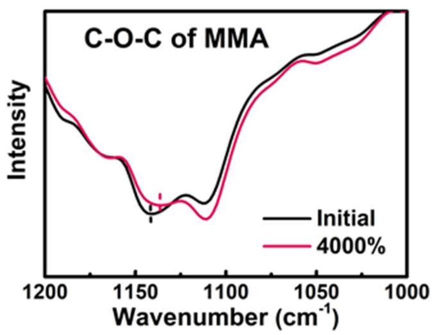

Supplementary Figure 17.

FTIR data for MEG2 (MMA:EGA=7:4, without lithium salts). The vibration frequency of C-O-C of MMA (1140 cm^{-1}) exhibited red shifts when the MEG2 specimen was stretched to 4,000%.

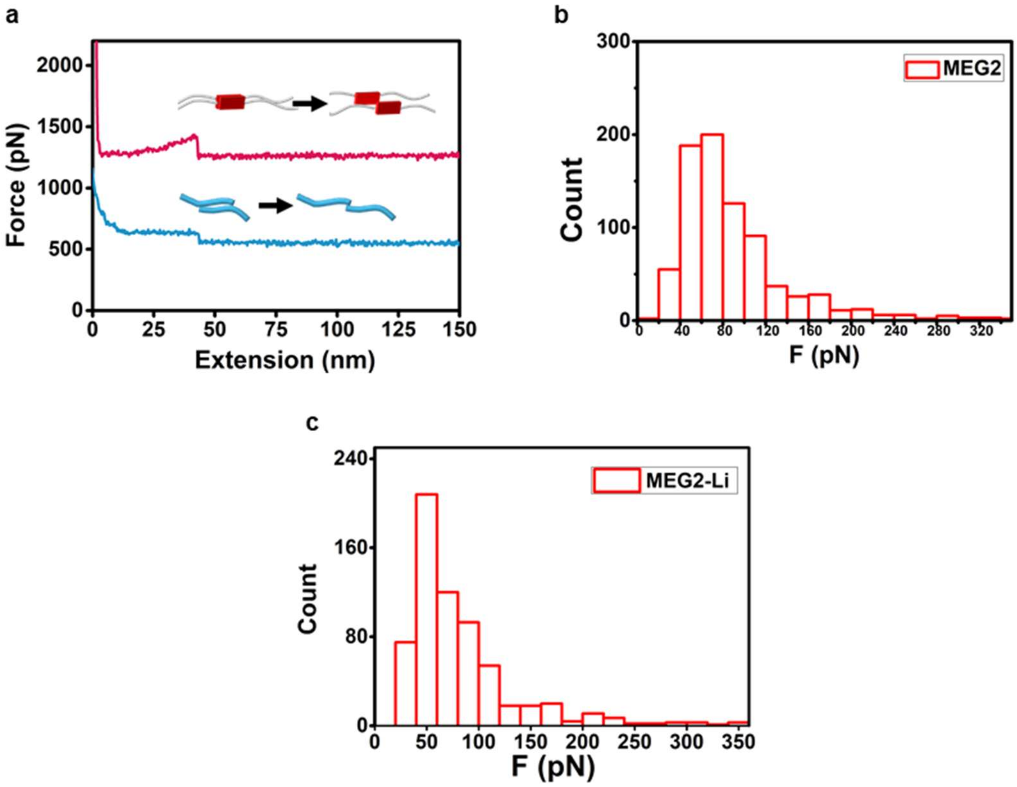

Supplementary Figure 18.

Single-molecule force spectrum. a, Characteristic force-extension curves for MEG2. **b,** Statistical Figure of force distribution for MEG2. **c,** Statistical Figure of force distribution for MEG2-Li.

	Ref.16	Ref.17	Ref.18	Ref.19	Ref.20	Ref.21
**Young's modulus (Mpa)**	0.375	0.83	0.0814	2.3	0.42	0.00166
**λ (%)**	17,100	8,100	15,100	10,100	10,700	10,100
	**Ref.22**	**Ref.23**	**Ref.24**	**Ref.25**	**Ref.26**	**This work**
**Young's modulus (Mpa)**	0.0273	1.71	0.7	0.037	0.028	18
**λ (%)**	5,700	18,100	13,500	21,100	11,800	30,100

Supplementary Table 1. Data statistic of "Young's modulus" and "λ" of stretchable materials whose stretchability is above 3,000% reported in literatures.

	**CSH-PPG-Zn-0.50 (23)**	**PF127 (26)**	**U-PDMS30K-E (22)**	**PI–43Na (24)**	**PU-BN7 (27)**
**Toughness (MJ/m^3)**	40.83	14.1	2.47	70	54.7
**λ (%)**	5,200	11,800	5,700	13,500	14,150
	**PU-BN9 (27)**	**PU-BN11 (27)**	**Poly(urea–urethane) (28)**	**CB[8] (20)**	**This work**
**Toughness (MJ/m^3)**	119.8	182.2	13	11	228
**λ (%)**	5,060	3,220	3,200	4,600	30,100

Supplementary Table 2.

Data statistic of "Toughness" and "λ" of stretchable materials whose stretchability is above 3,000% reported in literatures.

	Ligament(31)	Natural rubber (NR) (30)	Styrene butadiene Rubber (SBR) (30)	Isomerized natural rubber (INR) (29)	Synthetic Isoprene (SI) (30)
**Young's modulus (Mpa)**	0.1	2	1.6	2.5	1.7
**Threshold (J/m^2)**	1,000	40	60	70	70
	**Polychloroprene (PCP) (30)**	**Butadiene Acrylonitrile (BA) (29)**	**Composite silicone rubber (7)**	**This work**	
**Young's modulus (Mpa)**	1.6	2	0.1	18	
**Threshold (J/m^2)**	70	100	500	**2,682**	

Supplementary Table 3.

Data statistic of "Young's modulus" and "Threshold" on skeletal muscle and various rubbers.

	Young's modulus (MPa)	Maximum strength (MPa)	λ at break (%)
**MEG1-Li**	19.27 ± 0.51	1.55 ± 0.18	2,217 ± 164
**MEG2-Li**	17.57 ± 0.97	1.18 ± 0.086	30,000 without break
**MEG3-Li**	6.08 ± 1.39	0.30 ± 0.040	30,000 without break
**PMMA**	74.95 ± 0.44	5.04 ± 0.95	104 ± 0.81
**PEGA**	0.061 ± 0.0034	0.12 ± 0.016	280 ± 13
**MEG2**	14.42 ± 0.62	0.36 ± 0.029	30,000 without break

Supplementary Table 4.

Mechanical properties of MEG1-Li (MMA:EGA=2:1, Li^+:O=1:50), MEG2-Li (MMA:EGA=7:4, Li^+:O=1:50), MEG3-Li (MMA:EGA=3:2, Li^+:O=1:50), PMMA, PEGA and MEG2 (MMA:EGA=7:4).

	PMMA	PEGA	MEG2-Li
**Toughness (MJ/m^3)**	0.11 ± 0.0027	0.087 ± 0.0061	228.29 ± 24.87

Supplementary Table 5.

Toughness values of PMMA, PEGA and MEG2-Li (MMA:EGA=7:4, Li^+:O=1:50).

	MEG1-Li	MEG2-Li	MEG3-Li
**MMA:EGA**	2:1	7:4	3:2
**Li^{+}:O**	1:50	1:50	1:50
**Fracture energy (J/m^2)**	22,770±1,586	95,265±4,385	6,579±566

Supplementary Table 6.

Statistical data of fracture energy values for MEG1-Li, MEG2-Li, MEG3-Li.

	MEG1-Li	MEG2-Li	MEG3-Li	MEG2
**MMA:EGA**	2:1	7:4	3:2	7:4
**Li^+:O**	1:50	1:50	1:50	without lithium salts
**Fatigue threshold (J/m^2)**	1,736	2,682	2,360	612

Supplementary Table 7.

Fatigue thresholds of MEG1-Li, MEG2-Li, MEG3-Li and MEG2.

MEG2	Dipole-dipole interaction between PEGA		PMMA		Total
Type	Single plateau	Multiple plateaus	Big peaks	Big peaks and plateaus	
Force	40-60 pN	40-60 pN	120-240 pN	120-240 pN	
Ratio	38.74%	41.44%	12.61%	7.21%	
Number	215	230	70	40	555

Supplementary Table 8.

Statistical data for MEG2 (MMA:EGA=7:4, without lithium salts) by single-molecule force spectrum.

MEG2-Li	Dipole-dipole interaction between PEGA		Li^+-O ion-dipole interaction
**Type**	Single plateau	Multiple plateaus	Small peaks and multiple plateaus
**Force**	40-60 pN	40-60 pN	50-100 pN
**Ratio**	39.31%	24.53%	10.38%
**Number**	125	78	33
**MEG2-Li**	**PMMA**		**Total**
**Type**	Big peaks	Big peaks and plateaus	
**Force**	120-240 pN	120-240 pN	
**Ratio**	13.84%	11.95%	
**Number**	44	38	318

Supplementary Table 9.

Statistical data for MEG2-Li (MMA:EGA=7:4, Li^+:O=1:50) by single-molecule force spectrum.

Supplementary References.

1. M. Gordon. The physics of rubber elasticity (third edition). L. R. G. Treloar, clarendon press, oxford. *Polym. Int.* **8**, 39-39 (1976).
2. G. J. Lake, A. G. Thomas. The strength of highly elastic materials. *Proc. R. Soc. Lond., Ser. A. Math. Phys. Sci.* **108**, 300 (1967).
3. G. J. Lake, P. B. Lindley. The mechanical fatigue limit for rubber. *J. Appl. Polym. Sci.* **9**, 1233-1251 (1965).
4. S. Lin, J. Liu, X. Liu, X. Zhao. Muscle-like fatigue-resistant hydrogels by mechanical training. *Proc. Natl. Acad. Sci. U. S. A.* **116**, 10244-10249 (2019).
5. C. Xiang*, et al.* Stretchable and fatigue-resistant materials. *Mater. Today* **34**, 7-16 (2020).
6. X. Li*, et al.* Effect of mesoscale phase contrast on fatigue-delaying behavior of self-healing hydrogels. *Sci. Adv.* **7**, eabe8210 (2021).

7. M. W. Matsen, F. S. Bates. Unifying weak and strong segregation block copolymer theories. *Macromolecules* **29**, 1091 (1995).
8. J. F. Marko, E. D. Siggia. Stretching DNA. *Macromolecules* **28**, 8759-8770 (1995).
9. J. Paturej, S. S. Sheiko, S. Panyukov, M. Rubinstein. Molecular structure of bottlebrush polymers in melts. *Sci. Adv.* **2**, e1601478 (2016).
10. A. V. Dobrynin, J.-M. Y. Carrillo. Universality in nonlinear elasticity of biological and polymeric networks and gels. *Macromolecules* **44**, 140-146 (2011).
