# Supplementary material for: Superstretchable, yet stiff, fatigue-resistant ligament-like elastomers
Source: Nat Commun. 2022 Apr 27;13:2279. doi: 10.1038/s41467-022-30021-3 (PMC9046184; doi:10.1038/s41467-022-30021-3)
Supplement: Supplementary file 2 — Description of Additional Supplementary Files [file 41467_2022_30021_MOESM2_ESM.docx]

File name: Supplementary Information

**Description:**

Supplementary Discussions 1 to 7

Supplementary Figures 1 to 18

Supplementary Tables 1 to 9

Supplementary References 1 to 10

File name: Supplementary Movie 1.

**Description:** The process of MEG2-Li specimen (width:10 mm, thickness: 2.0 mm, initial length: 1.5 mm) being stretched to the stretch of 30,000% (0~29 s in movie). Even at extreme high stretch (30,000%), the MEG2-Li specimen was still remarkably elastic: it could come back from its stretched state to original state within 1 s (29 ~41 s in movie).

File name: Supplementary Movie 2.

**Description:** The MEG2-Li specimen (width:10 mm thickness: 2.0 mm) maintained its strong mechanical strength at 15,000% stretch, which could be used to lift a bucket of 1.5 kg alcohol.

File name: Supplementary Movie 3.

**Description:** The MEG2-Li square specimen with dimensions of 100 mm $\times$ 100 mm $\times$ 1.0 mm (L $\times$ W $\times$ T) could even bear the drop impact of 1.5 kg sharp conical hammer from the height of 0.1 m.

File name: Supplementary Movie 4.

**Description:** The MEG2-Li specimen (thickness: 1.8 mm) could even sustain a puncture from a sharp needle. After the punching, the rubbery materials quickly returned to its original state.

File name: Supplementary Movie 5.

**Description:** When we used the completely healed MEG2-Li specimen as the ligament and bent 180^o^. Impressively, the specimen could almost recover in less than 10 s, showing excellent elasticity at large scale even after healing.
